# Case Report: Restrictive cardiomyopathy due to a rare mutation in troponin I *gene* (*TNNI3*) in a patient

**DOI:** 10.3389/fcvm.2024.1456542

**Published:** 2024-11-20

**Authors:** Lili Deng, Liming Luo, Min Zhang, Cheng Guo, Kai Liu

**Affiliations:** ^1^ Department of Cardiology, Kunming Children’s Hospital, Kunming, Yunnan, China; ^2^ Department of Hematology, Kunming Children’s Hospital, Kunming, Yunnan, China; ^3^ Department of Otolaryngology, Kunming Children’s Hospital, Kunming, Yunnan, China; ^4^ Comprehensive Pediatrics, Kunming Children’s Hospital, Kunming, Yunnan, China

**Keywords:** *TNNI3* *gene*, mutation, restrictive cardiomyopathy, children, case

## Abstract

**Background:**

Restrictive cardiomyopathy (RCM) is a rare cardiomyopathy often characterized by normal or reduced ventricular chamber volume and bi-atrial enlargement, caused mainly by mutations in the myonodal gene. It has a low incidence, non-specific clinical manifestations, rapid progression, and lack of specific treatment, with heart transplantation usually being the ultimate treatment.

**Methods and results:**

This case reports a case of a 2-year-2-month-old boy located in Yunnan Province, China, who was admitted to the hospital with a 2-month history of orofacial bruising, aggravated by a 1-week history of bilateral eyelid swelling. After admission, electrocardiogram showed bi-atrial enlargement, echocardiography suggested bi-atrial enlargement with right and left ventricular diastolic hypoplasia, and cardiac magnetic resonance showed bi-atrial dilatation and possible localized myocardial fibrosis. A *de novo* heterozygous mutation (c.574C > T, p.Arg192Cys) in the *TNNI3* *gene* was identified by whole exome sequencing and verified by Sanger sequencing. The patient’s family opted for conservative treatment after diagnosis, but the patient died suddenly 2 months after diagnosis.

**Conclusion:**

This study identified a case of RCM due to TNNI3 mutation, emphasizing the importance of cardiac MRI and genetic testing in the clinical diagnosis of RCM and the need for heart transplantation. The study also revealed the possible heterogeneity of TNNI3 mutations across ethnic and geographic backgrounds, suggesting that long-term studies of genetic mutations should be strengthened in the future to promote the development of precision treatment strategies for cardiomyopathy.

## Introduction

Restrictive cardiomyopathy (RCM) is a rare and serious type of cardiomyopathy characterized by normal or reduced ventricular chamber volume and bi-atrial enlargement, which is often caused by myocardial ganglion gene mutations. The incidence of RCM is relatively low, accounting for only about 2.5%–3% of cardiomyopathies; the patients usually do not have specific clinical manifestations, but the disease progresses rapidly and lacks effective treatments, with heart transplantation usually being the ultimate treatment. Genetic studies have shown that myofibrillar gene variants are important pathogenic causes of RCM, and whole exome sequencing (WES) is a reliable method for identifying the causative genes of RCM. The most common myofibrillar gene variants associated with RCM include troponin T (TNNT2), α-cardiac troponin (ACTC), myosin-binding protein C54 (MYBPC3), tropomyosin 1 (TPM1), and myosin light chain 2 (TPM2), and myosin light chains 2 (MYL2) and 3 (MYL3), among others (1–4).

Although mutations in the above genes have been more frequently reported in Western populations, relatively few studies have been performed on Chinese and other East Asian populations. In recent years, it has been shown that the *TNNI3* *gene* is a susceptibility gene leading to hypertrophic cardiomyopathy (HCM) in East Asians, but its association with RCM has been less reported (5). In this case, we identified a rare mutation in the *TNNI3* *gene* by WES, further confirming the importance of genetic testing in the diagnosis of RCM. Through detailed clinical description and genetic analysis, we hope to provide new insights into the diagnosis and treatment of RCM.

We report a case of a 2-year-2-month-old boy from Yunnan Province, China, who was admitted to the hospital with orofacial bruising for 2 months, aggravated with bilateral eyelid swelling for 1 week. *De novo* heterozygous variants in the *TNNI3* *gene* (c.574C>T, p.Arg192Cys) were identified by WES and verified by Sanger sequencing. This case emphasizes the critical role of cardiac MRI and genetic testing in the clinical diagnosis of RCM and the importance of heart transplantation as the ultimate treatment. Meanwhile, this study examines the phenotypic heterogeneity of *TNNI3* *gene* mutations in different populations and suggests the need for future development of precision therapeutic strategies for RCM.

## Case report

A 2-year-old boy was admitted to the hospital with “bruising of the lips and mouth for more than 2 months, aggravated by swelling of both eyelids for 1 week.” Medical history denied any history of hypertension, diabetes mellitus, pericardial effusion, connective tissue disease, or radiation therapy, and there was no family history of sudden cardiac death or cardiomyopathy. He had been diagnosed with heart failure at an outside hospital 1 week earlier and was treated with diuretic therapy.

**Figure 1:**
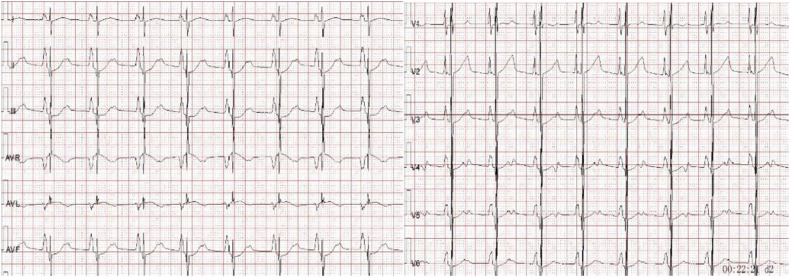
Electrocardiogram showing bi-atrial enlargement with lower and anterior wall ST-segment depression.

**Figure 2:**
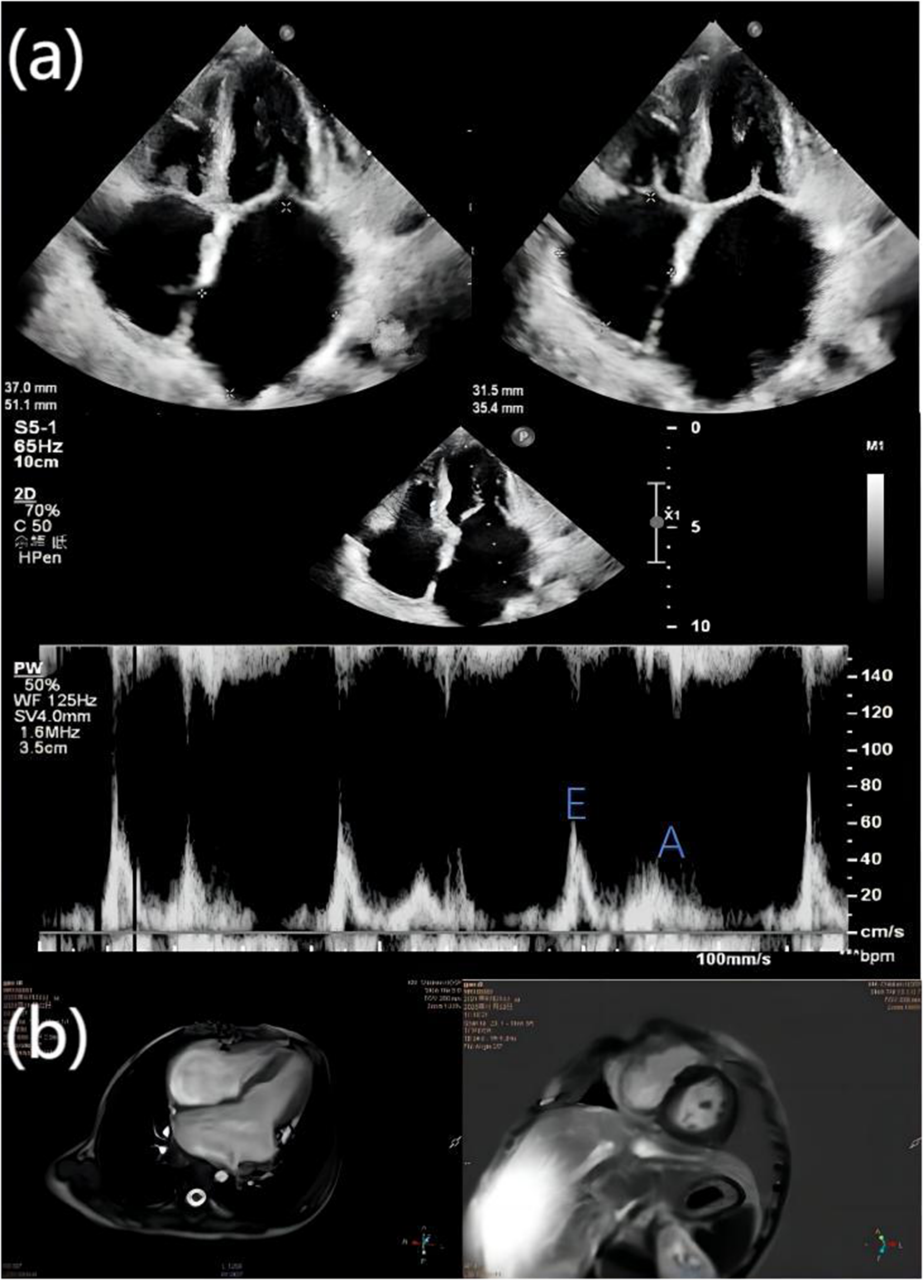
**(a)** echocardiography showed bi-atrial enlargement, left atrium diameter 37 mm × 51.1 mm, right atrium diameter 31.5 mm × 35.4 mm, right and left ventricular diastolic hypoplasia, normal septal and ventricular wall thickness, and trace pericardial effusion; the ratio of the peak E and peak A velocities of mitral inflow (E/A) was 2.05. The mitral inflow was 2.05%, and the peak A velocity was 2.05%. **(b)** The cardiac magnetic resonance four-chamber view of the heart shows bi-atrial enlargement and ventricular narrowing, and gadolinium-delayed enhancement in both chambers of the heart shows suspicious striations.

**Figure 3:**
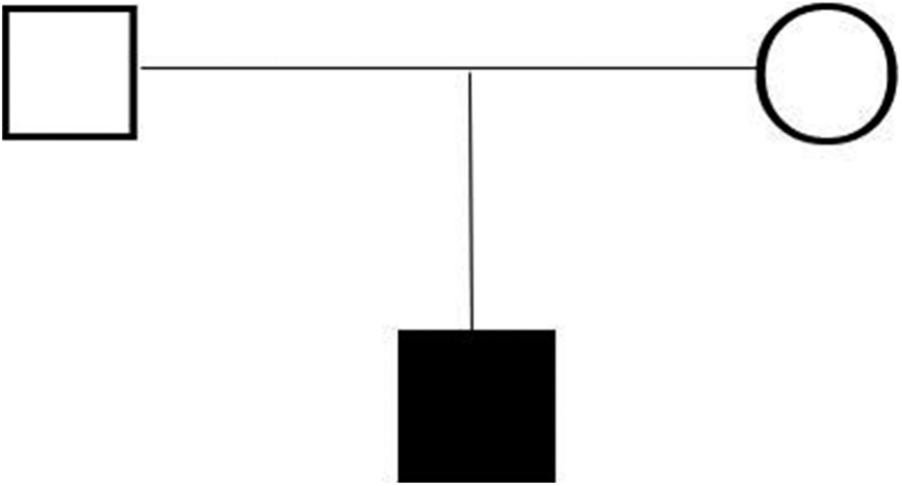
Family tree of the first witness (black fill). Circles: female, squares: male.

On admission, there was slight cyanosis of the lips and mouth, a little puffiness of the eyelids, jugular veins were full but not inflamed, heart rate was 110 beats/min, apical beat was slightly weak, but no obvious murmur was heard. The liver was moderately enlarged, and both lower limbs were mildly edematous. Electrocardiogram showed enlargement of both atria with depression of the ST segments of the lower and anterior walls (Figure 1). Echocardiography showed bi-atrial enlargement, left ventricular ejection fraction of 64.6%, normal septal and ventricular wall thickness, trace pericardial effusion, and no endocardial thickening. Mitral flow spectra showed an E/A of 2.05 (Figure 2). Cardiac magnetic resonance showed that the atrial and ventricular septum had uneven signals in the localized areas on perfusion imaging under resting state, and gadolinium-delayed enhancement showed a suspicious stripe enhancement of the septum (Figure 3). B-type natriuretic peptide was 1,050 pg/ml (normal < 100 pg/ml), and liver enzymes were mildly elevated. WES identified a heterozygous variant (TNNI3c.574C > T; p.Arg192Cys).

Based on the cardiac ultrasound results of the child, tricuspid valve peak E: 0.38, peak A: 0.49, E/A: 0.77 (normal range: <0.5–0.6 or >2.2–2.5), and right atrium diameter: 31.5 mm × 35.4 mm (horizontal × vertical diameters) indicated significant right atrial enlargement. Considering the above data, it was suggested that the child had reduced right ventricular diastolic function. During hospitalization, the doctor discussed with the family the possibility of performing a right heart catheterization to assess pulmonary vascular resistance for a better evaluation of the condition. Unfortunately, the family declined this examination due to concerns about the child's young age and the risks associated with right heart catheterization, resulting in a lack of data on pulmonary vascular resistance.

The following laboratory results were all negative or normal: creatinine and blood urea nitrogen levels, white blood cell count, urinalysis, antinuclear antibodies, antineutrophil cytoplasmic antibodies, erythrocyte sedimentation rate, gamma-interferon release assay, D-dimer level, HIV antibody level, and Anti-streptolysin O test.

Four candidate genes (*TNNI3*, *TNNI2*, *ACTC*, *MYH7*) known to be associated with restrictive cardiomyopathy were genetically analyzed by bidirectional sequencing of all coding exons. TNNI3 was found in affected children: NM000363.5:c.574C>T (p.Arg192Cys) (Supplementary Table S1). This missense mutation, previously described as associated with restrictive cardiomyopathy and hypertrophic cardiomyopathy, was verified by Sanger sequencing and was not detected in the peripheral blood of the parents, suggesting that it is *de novo* (Supplementary Table S1).

Analysis of exome sequencing data confirmed heterozygous TNNI3 mutations. The red arrow indicates the variant of P.Arg192Cys (c.574C > T).

Idiopathic RCM was considered to be the cause of this child's disease based on his medical history, cardiac ultrasound, cardiac magnetic resonance, and genetic testing, and he was treated with oral diuretics to reduce the volume load on the heart and to improve circulatory stasis. Two months after diagnosis, the child was rehospitalized due to influenza B infection and rapidly developed respiratory distress, consciousness impairment, and a progressively declining heart rate. Venous blood tests indicated B type natriuretic peptide (BNP) >5,000 pg/ml. Echocardiography showed an increase in pulmonary artery diameter from 14.7 to 16 mm, and tricuspid regurgitation estimated pulmonary artery pressure increased from 29 to 49 mmHg. A bedside chest X-ray revealed blurred pulmonary hilum. Despite resuscitation efforts, the patient was declared clinically deceased. The cause of death was considered to be respiratory failure and heart failure.

## Discussion

In our study, a pediatric case of RCM caused by a rare mutation in the *TNNI3* *gene* was reported in detail, and a *de novo* heterozygous variant of the *TNNI3* *gene* was identified by WES analysis. This finding has important implications for the diagnosis of idiopathic RCM. Although this mutation has been reported in other studies (6–8), our study provides a more detailed clinical description and genetic analysis, emphasizing the importance of cardiac MRI and genetic testing in the clinical diagnosis of RCM.

RCM is a type of cardiomyopathy characterized by severe diastolic dysfunction and restrictive filling. Its pathogenesis is complex, involving various molecular and cellular changes. The *TNNI3* *gene* is located on chromosome 19q13.42, with a total length of 6.2 kb, containing 8 exons and 7 introns, and encoding 210 amino acids. It is highly expressed in cardiac tissue. The cardiac troponin I (cTnI) encoded by the *TNNI3* *gene* is a key structural protein of cardiac muscle fibers, mainly regulating myocardial contraction and relaxation; mutations in the *TNNI3* *gene* are closely related to the development of RCM. At the molecular level, TNNI3 mutations cause RCM primarily by affecting the contractile and relaxation functions of cardiomyocytes. For example, the R170G/W mutations in the *TNNI3* *gene* are located in the regulatory C-terminal of cTnI, a region known to regulate the function of the inhibitory region of cTnI. Studies have shown that both mutations significantly enhance calcium sensitivity in removing membrane fibers, which is a typical characteristic of RCM mutations. In addition, compared to wild-type cTnI, these mutations significantly increase the affinity of troponin for actin, while their binding to tropomyosin is either enhanced (R170G) or reduced (R170W). Electron microscopy observations revealed that myofibrils containing R170G/W exhibited reduced stability, with undulations and breaks. These results suggest that the R170G/W variants of cTnI associated with RCM impair the communication between thin and thick filament proteins and disrupt the integrity of the thin filaments (9). At the cellular level, TNNI3 mutations may increase the sensitivity of myofilaments to calcium within cardiomyocytes, leading to a loss of normal relaxation ability during diastole, resulting in restricted ventricular filling, which is one of the core pathological mechanisms of RCM. For example, studies using induced pluripotent stem cell–derived cardiomyocytes (iPSC-CMs) have investigated the impact of TNNI3 mutations on myocardial relaxation, finding that specific TNNI3 mutations lead to impaired myocardial relaxation (10).

TNNI3 mutations have been shown to be associated with RCM and HCM. For example, a study by van den Wijngaard et al. found that TNNI3 mutations were associated with severe cardiomyopathy in the Dutch population, specifically with hypertrophic and restrictive cardiomyopathy (8). Pantou et al. described a case of RCM with a recessive inheritance pattern due to TNNI3 mutations in which the patient presented with a severe restrictive cardiomyopathy phenotype, and the condition among family members showed significant clinical heterogeneity (11). Kapoor et al. found that the D190Y mutation in TNNI3 was associated with RCM and was associated with mild cardiac hypertrophy in an Indian study, a finding that points to the fact that even the same genetic mutation may exhibit different pathologies in different populations (12).

Ishida et al. (13) used the Kaplan–Meier method and log-rank test to compare the overall survival probabilities of patients with or without pathogenic gene variants after being diagnosed with RCM. The 2- and 5-year survival rates for patients with pathogenic variants were 50% and 22%, respectively, while those without any candidate variants had survival rates of 62% and 54% (log-rank test; *P* = 0.0496). This indicates that patients with positive pathogenic variants had significantly poorer transplant-free survival compared to those without pathogenic variants.

An in-depth understanding of how mutations in the *TNNI3* *gene* affect cardiac troponin I function is essential for understanding the pathogenesis of RCM. Cardiac troponin I plays a key role in regulating contractile function in cardiomyocytes, and mutations may lead to protein dysfunction, which can trigger myocardial fibrosis and abnormal contractile function. The study by Sorrentino et al. extends our understanding of bi-allelic TNNI3 mutations in patients with early-onset dilated cardiomyopathy, showing that these mutations can trigger severe myocardial dysfunction (14).

The diagnosis and treatment of RCM faces many challenges. First, RCM is a rare and rapidly progressive cardiomyopathy for which there is no specific treatment, and heart transplantation is often the ultimate treatment. Our case emphasizes the importance of cardiac MRI and genetic testing in early diagnosis. Cardiac MRI provides detailed information about the structure and function of the heart, whereas genetic testing, particularly whole exome sequencing, can accurately identify disease-causing genetic variants.

Guidelines by Hershberger et al. emphasize the importance of genetic screening for cardiac troponin gene mutations, especially in patients with a family history of cardiomyopathy (15). Early detection of these genetic variants can provide patients with more targeted treatment options, which may improve their clinical prognosis.

In recent years, the multiple roles of TNNI3K in cardiac disease and its therapeutic possibilities have been investigated (16). Genetic variants in the human *TNNI3K* *gene* have been associated with supraventricular arrhythmias, conduction disease, cardiomyopathy, and sudden cardiac death.

Studies in mice have shown that TNNI3K is associated with cardiac hypertrophy, regeneration, and recovery after ischemia/reperfusion injury (14, 15, 17, 18). Positive effects on cardiac disease through inhibition of this kinase may delay the progression of heart failure and improve ventricular remodeling. Therefore, TNNI3K may be a potential target for the treatment of different cardiac diseases.

There are some limitations to our study. First, RCM is a rare and difficult-to-treat cardiomyopathy, and there are no established methods to replicate the typical phenotype of RCM *in vitro*, especially diastolic dysfunction. Second, TNNI3 mutations have a low prevalence in dilated cardiomyopathy, but our findings suggest that other recessive disease genes may be found by using molecular genetic strategies suitable for identifying homozygous sequence variants. In addition, more in-depth analysis of the causes of sudden death in children is needed to improve our understanding and treatment of RCM.

Overall, a detailed clinical and genetic analysis of the TNNI3 p.Arg192Cys mutation was performed in our case report, detailing the clinical presentation and family history of the patient, providing valuable clinical and genetic information for RCM research. Future research should enhance long-term studies of genetic mutations to better understand how these mutations affect the onset and progression of cardiomyopathies globally. In the meantime, long-term follow-up studies will help reveal the impact of these genetic mutations on the progression of cardiomyopathies as well as explore their potential impact on therapeutic strategies for cardiomyopathies.

## Conclusion

This case report of the p.Arg192Cys mutation in the *TNNI3* *gene* in Chinese children with RCM emphasizes the importance of genetic screening and early intervention. This study revealed phenotypic heterogeneity of this mutation in different populations, highlighting the role of environmental and genetic factors in the development of cardiomyopathy.

Future studies should enhance long-term research on genetic mutations through cross-ethnic and multicenter collaborations to advance the development of precision therapeutic strategies for cardiomyopathy.

## Data Availability

The datasets presented in this study can be found in online repositories. The names of the repository/repositories and accession number(s) can be found in the article/Supplementary Material.
