# Supplementary material for: Case Report: Restrictive cardiomyopathy due to a rare mutation in troponin I gene (TNNI3) in a patient
Source: Front Cardiovasc Med. 2024 Nov 20;11:1456542. doi: 10.3389/fcvm.2024.1456542 (PMC11614833; doi:10.3389/fcvm.2024.1456542)
Supplement: Supplementary file 1 [file Table1.docx]

| Gene | TNNI3 | Chromosomal  Location | chr19:55663261 | Variation  Information | c.574C>T  (p.Arg192Cys) |
| --- | --- | --- | --- | --- | --- |
| NP25FM0589  Index case Forward sequencing  Heterozygosis | | 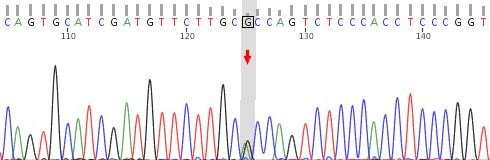 | | | |
| NP25FM0589  Index case Reverse sequencing  Heterozygosis | | 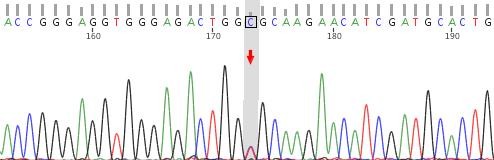 | | | |
| VP25D12512  Father Forward sequencing  Wild type | | 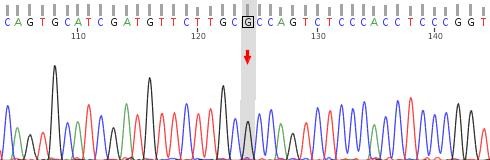 | | | |
| VP25D12511  Mother Forward sequencing  Wild type | | 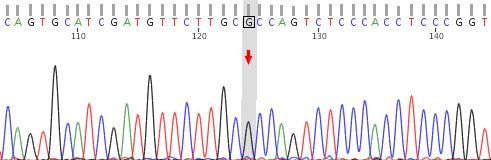 | | | |
